# HRAS Q61L Mutation as a Possible Target for Non-Small Cell Lung Cancer: Case Series and Review of Literature

**DOI:** 10.3390/curroncol29050300

**Published:** 2022-05-20

**Authors:** Laurent Mathiot, Guillaume Herbreteau, Siméon Robin, Charlotte Fenat, Jaafar Bennouna, Christophe Blanquart, Marc Denis, Elvire Pons-Tostivint

**Affiliations:** 1 Service d’Oncologie Médicale, CHU Nantes, Nantes Université, F-44000 Nantes, France; laurent.mathiot@etu.univ-nantes.fr; 2 UMR 1302/EMR6001, INCIT, Immunology and New Concepts in ImmunoTherapy, CNRS, INSERM, Laboratoire de Biochimie, CHU Nantes, Nantes Université, F-44000 Nantes, France; guillaume.herbreteau@chu-nantes.fr (G.H.); marc.denis@chu-nantes.fr (M.D.); 3 Pharmacie, CHU Nantes, Nantes Université, F-44000 Nantes, France; simeon.robin@chu-nantes.fr (S.R.); charlotte.fenat@chu-nantes.fr (C.F.); 4 Department of Medical Oncology, Hospital Foch, F-92073 Suresnes, France; j.bennouna@hopital-foch.com; 5 Nantes Université, Inserm UMR 1307, CNRS UMR 6075, Université d’Angers, CRCI2NA, F-44000 Nantes, France; christophe.blanquart@univ-nantes.fr

**Keywords:** non-small cell lung cancer, *HRAS* Gln61Leu, tipifarnib, oncogenic driver

## Abstract

Introduction: Assessment of actionable gene mutations and oncogene fusions have made a paradigm shift in treatment strategies of non-small cell lung cancer (NSCLC). *HRAS* mutations involved around 0.2–0.8% of NSCLC patients, mostly on codon 61. For these patients, few data are available regarding clinical characteristics and response to therapies. Methods: Next-Generation Sequencing (NGS) done routinely at Nantes University Hospital was used to identify *HRAS* molecular alterations in NSCLC patients. We identified and described four *HRAS* p.GlnQ61Leu mutated patients. Literature of previously *HRAS*-mutant NSCLC cases was reviewed, and available data in solid tumour with the most advanced H-Ras specific inhibitor, tipifarnib, were presented. Results: Of 1614 patients diagnosed with advanced NSCLC from January 2018 to December 2020, four (0.25%) had *HRAS* p.Gln61Leu mutation. Three of them died during the first-line systemic therapy. Furthermore, three additional cases were identified in literature. All cases were current or former smokers, most of them had pleural or pericardial effusion at diagnosis. Conclusions: The clinical course of patients with *HRAS*-mutant NSCLC remains unclear. Furthers cases should be identified in order to clarify prognosis and response to therapies. Tipifarnib, a farnesyl transferase inhibitor, is a promising candidate to target *HRAS*-mutant tumours and should be explored in NSCLC patients.

## 1. Introduction

Lung cancer accounted for more than 2.2 million cases and almost 1.8 million deaths worldwide in 2020 [1]. Non-small cell lung cancer (NSCLC) is the main type of lung cancer and represents 85% of all cases. The prognosis of oncogenic-addicted NSCLC has been transformed due to the development of targeted therapies, firstly inhibiting the mutant epidermal growth factor receptor (*EGFR*) [2,3], then *ALK* [4,5,6] and *ROS1* rearrangement [7]. More recently, *KRAS* appeared as a promising target in NSCLC patients, due to the development of efficient targeted therapies such as sotorasib targeting the *KRAS* p.Gly12Cys mutation [8,9,10].

The *RAS* genes belong to a well-described family of oncogenic drivers, encoding four small GTPase proteins, K-Ras4A and K-Ras4B (which are two splice variants of the *KRAS* gene), H-Ras and N-Ras [9,10,11]. These proteins have an active form, Ras-GTP, and an inactive form, Ras-GDP. Transition from one to another is regulated by Ras-Guanine nucleotide Exchange Factors (Ras-GEFs) that catalyse the exchange of GDP for GTP, and by intrinsic GTPase activity hydrolysing GTP to GDP with the assistance of Ras-GTPase Activating Proteins (Ras-GAPs). In humans, Ras proteins participate in many physiological processes related to cell growth, division, and survival [10]. Substitution mutations, mainly involving codons Gly12, Gly13, and Gln61 of the *RAS* genes, are found in about 20% of human cancers. They are known to cause constitutive activation of the signalling activity mainly through impairment of their intrinsic GTPase activity [12,13] and thus of the conversion of active form Ras-GTP to inactive form Ras-GDP. Ras-GTP interact with downstream effectors such as PhosphoInositide 3-Kinase (PI3K) and Mitogen Activated Protein Kinases (MAPK) pathways, that enhanced growth, proliferation, differentiation, and survival of cancer cells [10].

Based on a recent publication evaluating several leading cancer mutation databases, *KRAS* is by far the most commonly mutated of the three *RAS* genes in solid tumours (75%), followed by *NRAS* (17%) and *HRAS* (7%) [13]. *HRAS* is mostly altered in head and neck squamous cell carcinoma (HNSCC) (5–9%), salivary glands (15%), and bladder cancer (5–30%) [13,14]. *HRAS* mutations are involved around 0.2–0.8% of lung adenocarcinoma and squamous cell lung cancer patients [13]. Among *HRAS* mutated tumours, single-base substitutions mutations occurring most frequently on codon 12 (27–33%), 13 (25–27%), or 61 (37–40%) [13,15,16].

In this study, we reported the incidence of *HRAS* mutations among a large cohort of NSCLC patients, and we focused on the clinical-pathological features of four cases with *HRAS* p.Gln61Leu mutations. Then, we completed with literature review of previously described *HRAS*-mutant NSCLC cases. Last, we described available data in solid tumours (lung cancer and others) with the most advanced H-Ras specific inhibitor, tipifarnib.

## 2. Patients and Methods

A multicentre retrospective study was performed including all newly diagnosed NSCLC patients between 1 January 2018 and 31 December 2020 with their genomic analyses performed at Nantes University Hospital. Next-generation sequencing (NGS) was systematically performed for all newly diagnosed non-squamous NSCLC patients presenting advanced disease, and never smokers squamous NSCLC patients. Briefly, DNA was extracted from formalin-fixed paraffin-embedded (FFPE) tumours using the Maxwell RSC RNA FFPE kit (Promega, Madison, WI, USA). NGS libraries were synthesized using the QIAseq Targeted DNA Custom Panel (QIAGEN, Hilden, Germany) kit, an amplicon library construction kit based on Anchored Multiplex PCR (AMP) technology. Sequencing was performed on a MiSeq sequencer (Illumina, San Diego, CA, USA), and NGS data analysis was performed using the Biomedical Genomics Workbench (QIAGEN). Panel of tested genes is shown in the Table A1. Patients with *HRAS*-mutant tumours were identified, and clinical data from the medical records of *HRAS* p.Gln61Leu mutated patients (the most frequently observed *HRAS* mutation) were extracted. Other cases included in the literature were reviewed from Medline database, using the keywords “non-small cell lung cancer” and “HRAS”. This study was conducted in accordance with the local regulations, and it was approved by the local independent ethics committee.

## 3. Results

### 3.1. Cases Presentation

**Figure 1:**
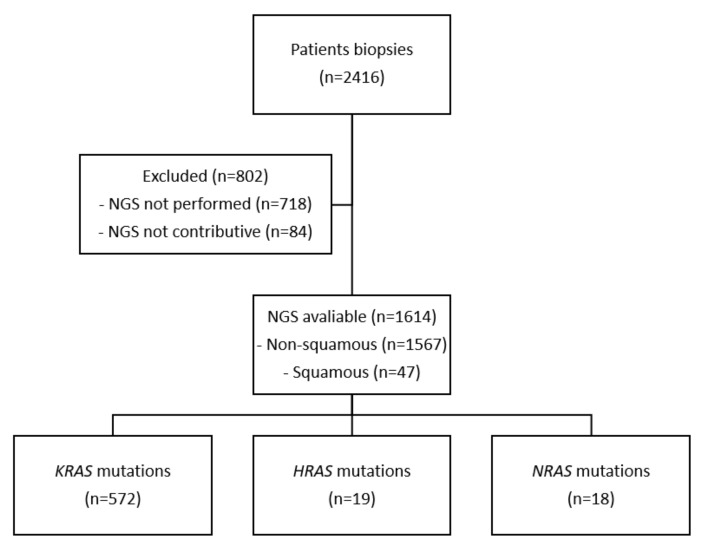
Flowchart. NGS: Next-Generation Sequencing.

NGS was performed on 1614 patients during recruitment (Figure 1). Nineteen (1.18%) had *HRAS* mutation, 572 (35.43%) had a *KRAS* mutation, and 18 (1.12%) had an *NRAS* mutation. The mutation most frequently observed was *HRAS* p.Gln61Leu, in four patients (Table 1). All cases are reported in Table 2.

The first one was an active smoker 50-year-old female, with a past medical history of hypertension and chronic obstructive pulmonary disease. In 2015, she underwent right lobectomy and adjuvant chemotherapy (cisplatin and pemetrexed) for a pT4N0M0 lung adenocarcinoma. In January 2019, a control computed tomography (CT) scan revealed an upper left lobe mass, mediastinal adenopathies, liver metastases, and pericardial effusion. She had no symptoms at this time. Histology of pericardial effusion after drainage showed lung adenocarcinoma cells, Tumour Proportion Score (TPS) PD-L1 < 1%. NGS showed an *HRAS* p.Gln61Leu mutation (variant allele fraction (VAF) 10.0%) with no other alteration. She started a first-line chemotherapy with carboplatin and pemetrexed; however, she only received three cycles of chemotherapy due to a major cachexia. A CT scan confirmed a lung, liver, and bone progression. She died four weeks later.

The second case was a 55-year-old male, with a past medical history of pulmonary embolism and diabetes mellitus. He was a former smoker of around 60 packs per year. Lung cancer was diagnosed in July 2019. The histology revealed a Not Otherwise Specified (NOS) carcinoma, TTF1 and P40 negative, TPS PD-L1 = 0%. NGS showed *KRAS* p.Gly12Cys (VAF 19.6%) and *HRAS* p.Gln61Leu mutations (VAF 27.9%). CT showed a right upper lobe mass with bronchus invasion, bilateral mediastinal and hilar lymph node invasion, with no extra-thoracic extension. Health status was preserved with a performance status (PS) score of 1. He initiated a first-line chemotherapy with four cycles of carboplatin and pemetrexed, followed by maintenance with pemetrexed. He was hospitalized one month after the beginning of maintenance therapy for altered general condition. CT revealed disease progression, and the patient died one month after.

The third case was an active smoker 63-year-old male, 35 packs per year. His only antecedent was hypertension. He was diagnosed in August 2020, in a context of headache, dizziness, and asthenia. CT showed three brain metastases, a 67mm right mediastino-hilar mass, multiple mediastinal adenopathies, and a pericardial effusion. Histology revealed a NOS carcinoma, TPS PD-L1 = 60%. NGS showed *KRAS* p.Gly12Ser (VAF 27.7%), *TP53* c.784_809del (VAF 26.45%) and *HRAS* p.Gln61Leu mutations (VAF 29.1%). A whole brain radiotherapy of 30 Gy was performed leading to a neurological improvement. He was treated with a combination of chemotherapy plus immunotherapy (carboplatin, pemetrexed and pembrolizumab) on September 2020. He received four cycles followed by monotherapy with pembrolizumab. The first evaluation in December 2020 showed a partial response of 39%. The patient was still treated at the time of data closure in November 2021.

The fourth case was a 61-year-old female, without past medical history. She was a highly active smoker with 68 packs per year. The presence of a dysphonia led to a chest CT in September 2020 and revealed a large compressive mediastino-hilar mass, and moderate abundance of bilateral pleural effusion. A brain CT and a PET-CT showed no extra-thoracic metastases. Histology was an adenocarcinoma, TPS PD-L1 = 60%. NGS showed *TP53* p.Ile195Thr (VAF 13.6%) and *HRAS* p.Gln61Leu mutations (VAF 28.1%). She was hospitalized for obstructive pneumopathy, atrial fibrillation, and cardiogenic failure. She died in November 2020.

### 3.2. Review of Literature

To our knowledge, only three other individual clinical cases of NSCLC patients with *HRAS*-mutant tumours have been described in literature. All cases were p.Gln61Leu mutations (Table 2). According to the TCGA database (pro-jects TCGA-LUAD and TCGA-LUSC), *HRAS* mutations were found in 3/550 (0.55%) adenocarcinomas and 8/480 (1.67%) squamous cell carcinomas (data available online: https://portal.gdc.cancer.gov/ accessed on 14 April 2022). One adenocarcinoma had *HRAS* p.Gln61Leu mutation. The first case was from the USA, and the two others came from China. The first patient was a former smoker 79-year-old male, with a rapid progression from stage IB disease to metastatic adenocarcinoma and death [17]. His only antecedent was meningioma. The patient underwent right upper lobectomy in September 2010, for a localized pT2aN0M0 adenocarcinoma. He received four cycles of adjuvant carboplatin and pemetrexed. He relapsed 10 months after the surgery, with brain metastases, multiple bone, liver, and bilateral adrenal lesions. He underwent craniotomy for a temporal lobe mass and stereotaxic radiosurgery of additional brain lesions. The molecular biology of the brain lesion revealed a *HRAS* p.Gln61Leu mutation. Other mutations, such as *KRAS*, *TP53*, and 51 other genes were negative. He was unable to receive chemotherapy and died in January 2012.

The second patient was a 58-year-old male without past medical history, who showed rapid metastatic progression and passed away due to respiratory failure after 2 weeks of systemic treatment [18]. He was an active smoker. He was diagnosed with a cavitary nodule of the right lung and malignant pleural effusion. Histology suggested a poorly differentiated carcinoma. Video-assisted thoracoscopic lobectomy and lymph node dissection was performed for palliative symptoms management. Amplification Refractory Mutation System (ARMS)- PCR assay showed a weak signal for p.Leu858Arg and p.Thr90Met of *EGFR* mutation, thus he was treated with cisplatin and osimertinib. He died 15 days after. A reassessment of the primary tumour and lymph nodes showed *HRAS* p.Gln61Leu and *NRAS* p.Gln61Lys mutations, but *EGFR* mutations were not detected.

A third case, although from China, was reported. It was a 76-year-old man with an antecedent of Lynch syndrome and 50-year smoking history [19]. Chest CT showed tumour masses in the upper lobe and hilar of right lung, with mediastinal lymphadenopathies, presenting no other distant metastases on PET-CT. Histology identified a squamous cell carcinoma, TPS PD-L1 = 50%. NGS showed *HRAS* p.Gln61Leu and *TP53* p.Arg158Leu. Other genes such as *LRP1B, DNMT3A*, *POLE, TCF7L2,* or *NTM* were also mutated. A heterozygous germline mutation of *MSH6* was detected, but immunohistochemistry suggested normal expression of MLH1, MSH2, MSH6, and PMS2 in tumour biopsy. He was treated with pembrolizumab and achieved stable disease as the best response. Disease progression finally occurred after eight cycles. At this moment, plasma circulating tumour DNA test revealed a *STK11* mutation, which was not present at diagnosis. Subsequently, the patient received chemotherapy with albumin-bound paclitaxel.

## 4. Discussion

Among non-squamous NSCLC, while *KRAS* mutations account for around 30% of patients, *HRAS* and *NRAS* mutations account for less than 1% [13,20,21]. Concordant with previous studies, we found a low prevalence of 0.82% *HRAS* mutations among NSCLC patients (19/1614 patients). In our centre, according to national guidelines, NGS was not routinely performed in squamous cell carcinoma, excepted for exceptional never-smokers. Thus, we might have missed other *HRAS*-mutant tumours, since *HRAS* mutations were also rarely described in squamous cell carcinoma [13,22]. A dedicated study in this population should be performed.

The clinical course of a patient with *HRAS*-mutant NSCLC remains unclear. Indeed, we have found only three cases describing *HRAS*-mutant lung cancer patients, all of them being *HRAS* p.Q61L. Our four cases plus the three others described in literature found that all patients were active or former smokers. Pleural and/or pericardial effusion was described in four out of seven cases. Clinical presentation was mostly aggressive, with one newly diagnosed patient who died before any treatment and four others who died during first-line treatment. Two patients received immunotherapy: one was still under treatment after more than one year, and the other received eight cycles with a stable disease as the best response.

**Figure 2:**
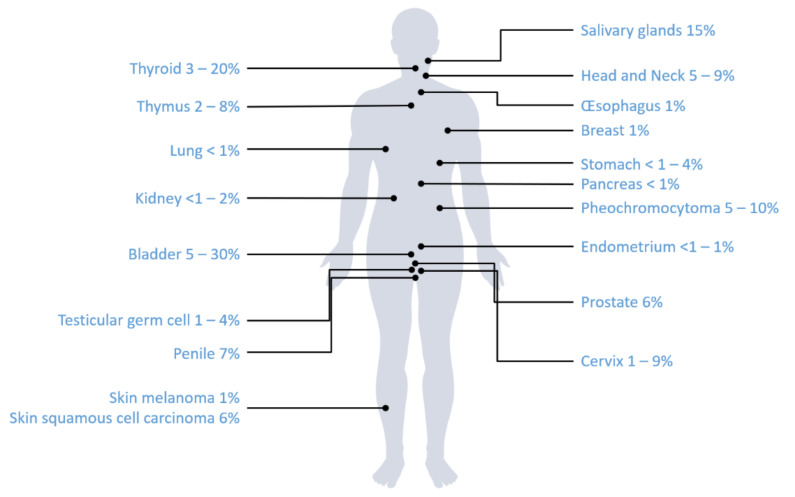
Frequencies of *HRAS* mutations among solid cancer types.

*HRAS* mutations have been mainly documented in HNSCC, salivary glands, pheochromocytoma and bladder cancers [10,13,14,20,21,23,24,25,26,27,28,29] (Figure 2). *HRAS* mutations were known to be oncogenic; it was demonstrated in vitro that mutant *HRAS* hyperactivated MAPK and mTOR pathways in various cancer cell lines including lung, bladder, and oesophageal cancer [30,31]. Thus, it might appear as a potential interesting target. Among therapeutic strategies targeting *HRAS*-mutant cancers, tipifarnib, a farnesyl transferase (FT) inhibitor, is one of the most promising candidates [16,32]. Considerable work on FT inhibitors have been performed since 20 years, but these treatments failed to demonstrate any benefit in unselected populations, ending the development of these treatments as pan-Ras-targeted strategy [33]. Farnesylation is a mechanism needed for RAS molecules to be integrated in plasma membranes and for its activation. Contrary to tyrosine kinase inhibitors (TKI) such as EGFR TKIs that directly inhibit enzyme, tipifarnib indirectly disrupts RAS activity through preventing appropriate intracellular localization by interfering with RAS prenylation [31]. Although all RAS isoforms are FT substrates, only H-Ras exclusively depends on farnesylation for its membrane localization. Indeed, prenylation of H-Ras mostly depends on farnesylation, whereas K-Ras (4A and 4B) and N-Ras can be integrated in plasma membranes via other mechanisms, such as geranylgeranylation [16,34]. Accordingly, blockade of FT is sufficient to inhibit the action of H-Ras. Due to the development of *RAS* mutations profiling, tipifarnib appeared as an interesting strategy to inhibit *HRAS*-mutant tumours [33]. Its indirect mechanism of action is very different from covalent KRAS inhibitors (such as sotorasib and adagrasib), which act by selectively forming a covalent bond with cysteine 12 within the switch-II pocket of KRAS-G12C protein, thereby locking KRAS in the inactive state to arrest cell proliferation [35].

Importantly, 2/4 patients with *HRAS* mutation in our cohort also had a co-occurring *KRAS* mutation on codon 12. As *KRAS* mutations are described as strong oncogenic mutations, it remains unclear whether *HRAS* is a driver mutation or a passenger one and if those patients would benefit from *HRAS* inhibition.

Tipifarnib activity was characterized in a wide panel of *HRAS*-mutant and wild-type HNSCC xenograft models, and also in HNSCC patient-derived xenografts [31]. Considering the rarity of this mutation in lung cancer, extensive pre-clinical approach seems difficult to assess. However, using CRISPR-CAS 9 technology, *HRAS* mutation might be introduced in an appropriate lung cancer cell line in order to mimic the clinical situation. It is important to highlight that most of the activating *HRAS* mutations involved codon 12, 13, 61, 117, and 146. To our knowledge, other mutations have not been characterized in NSCLC. Nonetheless, tipifarnib seemed efficient regardless of *HRAS* hotspot mutation, as it inhibited activity on patient derived xenograft HNSCC models harbouring Q61L, G12C, G12S, G13R, and K117N mutations [31].

We resumed published clinical trials evaluating tipifarnib in solid tumours in Table 3 [36,37,38,39,40,41,42,43,44]. Only two were specifically conducted in lung cancer, with no selection based on *HRAS* status. The first clinical trial was carried out in 2003 [43]. This phase II study tested the tipifarnib in first-line setting of 44 patients with a locally-advanced NSCLC, not amenable to chemo-radiotherapy. No objective responses were documented, whereas inhibition of farnesylation in vivo was consistently documented. Median time to progression was 2.7 months, and seven patients (16%) had disease stabilization for greater than 6 months. Tipifarnib was also tested in 22 patients with sensitive relapsed small-cell lung cancer in a phase II trial [42]. No significant antitumour activity was observed. Median progression-free survival was only 1.4 months. 

In 2021, a phase II study enrolling 30 patients with recurrent and/or metastatic HNSCC harbouring *HRAS* mutation shown highly promising results. Objective response rate was 55% (11/20 patients; 95% confidence interval (CI), 31.5 to 76.9), with a median progression-free-survival of 5.6 months (95% CI, 3.6 to 16.4), and a median overall survival of 15.4 months (95% CI, 7.0 to 29.7) [36]. The most frequent adverse events were anaemia (37%) and lymphopenia (13%). The mechanistic basis of tipifarnib toxicity might be related to the inhibition of several dozen other farnesylated proteins in cells [45]. Moreover, tipifarnib was recently described as an inhibitor of the CXCL12/CXCR4 pathway, that might lead to other side effects [46]. A phase II study evaluating tipifarnib in *HRAS*-mutant squamous NSCLC (NCT03496766) and another phase II study of tipifarnib in HNSCC with *HRAS* mutations are ongoing (NCT02383927).

## 5. Conclusions

*HRAS* mutations are uncommon genomic alterations in NSCLC patients, representing less than 1% of patients. Further studies are needed to better understand clinical course and prognosis associated with these mutations. However, preliminary data seems to show an association with tabagic status, aggressive presentation, and co-occurring mutations in MAPK pathway and TP53. Interestingly, a targeted therapy against *HRAS*-mutant showed promising results in HNSCC patients. Whether *HRAS* could represent a druggable oncogene in NSCLC is still unknown and should be explored in further studies.

## Figures and Tables

**Table 1:** *HRAS* mutations type among all 19 patients.

*HRAS* Mutations	Numbers of Patients
p.Q61L (p.Gln61Leu; C.182A>T)	4
p.G13V (p.Gly13Val; c.38G>T)	2
p.E98K (p.Glu98Lys; c.292G>A)	1
p.S89F (p.Ser89Phe; c.266C>T)	1
p.A11P (p.Ala11Pro; c.31G>C)	1
p.K117N (p.Lys117Asn; c.351G>T)	1
p.R102L (p.Arg102Leu; c.305G>T)	1
p.D107fs (p.Asp107fs; c.319del)	1
p.V109L (p.Val109Leu; c.325G>T)	1
p.T58I (p.Thr58Ile; c.173C>T)	1
p.T148P (p.Thr148Pro; c.442A>C)	1
p.R41W (p.Arg41Trp; c.121C>T)	1
p.R135Q (p.Arg135Gln; c.404G>A)	1
p.M72I (p.Met72Ile; c.216G>T)	1
p.E76D (p.Glu76Asp; c.228G>T)	1

**Table 2:** Clinico-pathologic features of non-small cell lung cancer patients with *HRAS* p.Gln61Leu mutation reported in literature. ADC: adenocarcinoma; SCC: squamous cell carcinoma; ADSQ: adenosquamous; NOS: not otherwise specified; WBRT: whole brain radiotherapy; PFS: Progression-Free-Survival; OS: Overall Survival; NA: Not Available.

Reference	Sex (Female/Male)	Age at Diagnosis (Years)	Smoking Status	Pathology	PD-L1 (%)	Other Alterations	Metastatic Site	Treatment	PFS (Weeks)	OS (Weeks)
Current	F	50	Active	ADC	<1	None	Lung/Liver/Pericardial effusion (4 years after adjuvant chemotherapy)	Carboplatin—Pemetrexed	11	15
Current	M	55	Former	NOS	<1	KRAS p.Gly12Cys	Locally-advanced disease	Carboplatin—Pemetrexed	22	30
Current	M	63	Active	NOS	60	KRAS p.Gly12Ser TP53 c.784_809del	Brain/Pericardial effusion	Carboplatin—Pemetrexed—Pembrolizumab WBRT	On treatment	On treatment
Current	F	61	Active	ADC	60	TP53 p.Ile195Thr	Pleural effusion	Not treated	NA	3
Cathcart-Rake E., 2014 [17]	M	79	Former	ADC	NA	None	Brain/Bone/Liver/Adrenal (10 months after adjuvant chemotherapy)	Adjuvant Carboplatin—Pemetrexed Brain surgery—Stereotactic radiosurgery	NA	64
Zhao J., 2021 [18]	M	58	Active	ADSQ	NA	EGFR p.Leu858Arg and p.Thr790Met (only on pleural effusion), NRAS p.Gln61Lys	Pleural effusion	Cisplatin—Osimertinib	2	4
Long Y., 2021 [19]	M	76	Active	SCC	50	TP53 p.Arg158Leu LRP1B p.Val3711Phe LRP1B p.Leu4013Met DNMT3A p.Wrp601 * DDR2 p.Val336Leu NTM p.Thr240Ile TCF7L2 p.Arg420Pro POLE p.Arg579Leu	Locally-advanced disease	Pembrolizumab	24	NA

* is used for stop codon in HGSV nomenclature.

**Table 3:** Clinical trials evaluating Tipifarnib in solid tumours. HNSCC: Head and Neck Squamous Cell Carcinoma; NSCLC: Non-Small Cell Lung Cancer; SCLC: Small Cell Lung Cancer; NGS: Next-Generation Sequencing; PO: Per Os; ORR: Overall Response Rate; SD: Stable Disease; PFS: Progression Free Survival; OS: Overall Survival; VAF: Variant Allele Frequency; NA: Not Applicable.

Reference	Phase	Tumour Site	Number of Patients	Setting	Biomarker	Tipifarnib Dose & Schedule	Primary Endpoint	ORR (%) (SD)	Median PFS (Months)	Median OS (Months)
Ho A.L., 2021 [36]	2	HNSCC	22	Relapsed	Missense *HRAS* mutation/VAF > 20% either in blood, primary tumour tissue, recurrent or metastatic disease	800 or 900 mg PO twice daily on days 1–7 and 15–21 of 28-day cycles	ORR	50 (41)	5.6	15.4
Haddad R., 2021	2 (Ongoing)	HNSCC	NA	Relapsed	R/M m*HRAS* VAF ≥ 20% (tumour tissue) detected by NGS	600 mg PO with a meal twice a day for 7 days in alternating weeks (Days 1–7 and 15–21) of 28-day cycles	ORR in High VAF population	55 (NA)	NA	15.4
Hanna G.J., 2020 [35]	2	Salivary gland carcinoma	13	Relapsed	Missense *HRAS* mutation with a VAF > 20%: 54% p.Gln61Arg (tumour tissue)	900 mg PO twice daily on days 1 to 7 and days 15 to 21 of a 28-day	ORR	8 (54)	7.0	18.0
Lee H.W., 2020 [36]	2	Urothelial carcinoma	21	Relapsed	Missense, nonsynonymous *HRAS* mutations (p.Gly13Arg, *n* = 7; p.Gln61Arg, *n* = 4; p.Gly12Ser, *n* = 3; p.Gly12Cys, *n* = 2) (tumour tissue)	900 mg PO twice daily on days 1–7 and 15–21 of 28-day	6-month PFS	24 (62)	4.7	6.1
Jazieh K., 2019 [37]	1	Advanced, recurrent or metastatic solid tumours	27	Relapsed	No selection on *HRAS* status Tumour tissue (diagnostic)	4 dose levels, ranging from tipifarnib 200 mg PO twice daily plus erlotinib 75 mg PO once daily to tipifarnib 300 mg PO twice daily plus erlotinib 150 mg PO once daily	Safety, tolerability, maximum tolerated dose	7.4 (37)	NA	NA
Whitehead R.P., 2006 [38]	2	Metastatic colorectal adenocarcinoma	62	No prior chemo: 33/55 Prior chemo: 22/55	No selection on *HRAS* status Tumour tissue (diagnostic)	Fixed dose of 300 mg PO, twice daily, immediately after a meal, days 1–21, every 28 days, until tumour progression or toxicity	Confirmed response probability	2 (20)	1.7	8.1
Lara Jr P.N., 2005 [39]	1	Advanced, recurrent or metastatic malignant tumours: (8) NSCLC, (6) colorectal, (3) prostate, (1) oesophagial, (1) pancreatic, (1) parotid, (1) renal	21	Relapsed	No selection on *HRAS* status Tumour tissue (diagnostic)	Starting dose was 300 mg PO twice daily with escalation by 300 mg. Increments over six dose levels to a maximum of 1800 mg PO twice daily, on days 1–7 and 15–21 of 28-day treatment cycles	Not mentioned	0 (32)	NA	NA
Heymach J.V., 2004 [42]	2	SCLC	22	Relapsed	Missense *HRAS* mutation	3-week cycles at a dose of 400 mg PO twice daily for 14 consecutive days followed by 7 days off treatment	ORR	0 (5)	1.4	6.8
Adjei A., 2003 [43]	2	NSCLC	44	100% No prior chemotherapy (eligibility criteria) 9/44 had radiotherapy	No selection on *HRAS* status	300 mg PO twice daily for 21 of every 28 days	ORR	0 (16)	2.7	7.7
Hahn S., 2002 [44]	1	NSCLC	9	No prior therapy: 7/9 Prior chemotherapy: 2/9	No selection on *HRAS* status Tumour tissue (diagnostic)	Dose-escalation study of tipifarnib Dose level, 280 mg/m2 daily PO during weeks 1, 2, 4, and 5 of radiotherapy Dose level, 560 mg/m2 daily during weeks 1, 2, 4, 5, and 7 of radiotherapy	Maximum tolerated dose, dose limiting toxicity	NA	NA	NA

## Data Availability

The data presented in this study are available on request from the corresponding author.

## Appendix A

**Table A1:** Next-Generation Sequencing (NGS).

Sequenced Regions
AKT1 exon 3 (NM_001014431.1)
ALK exons 22 to 25 (NM_004304.1)
BRAF exons 11 and 15 (NM_004333.4)
CTNNB1 exon 3 (NM_001904.4)
DDR2 exons 5 to 10 and 14 to 19 (NM_001014796.2)
EGFR exons 18 to 21 (NM_005228.3)
ERBB2 (HER2) exons 19 to 22 (NM_004448.2)
ERBB4 codons 393 and 452 (NM_005235.2)
FGFR2 codons 252, 549 and 659 (NM_000141.4)
FGFR3 exons 6, 8 and 13 (NM_000142.4)
*HRAS* exons 2, 3 and 4 (NM_005343.2)
IDH1 codons 100 and 132 (NM_005896.3)
IDH2 codon 172 (NM_002168.3)
KIT exons 8, 9, 11, 13, 14, 17 and 18 (NM_000222.2)
KRAS exons 2, 3 and 4 (NM_033360.2)
MAP2K1 (MEK1) exon 2 (NM_002755.3)
MET exon 2, intron 13 and exons 14 to 20 (NM_001127500.1)
NRAS exons 2, 3 and 4 (NM_002524.3)
PDGFRA exons 12, 14 and 18 (NM_006206.4)
PIK3CA exons 10 and 21 (NM_006218.2)
RET exons 11 and 16 (NM_020975.6)
TP53 exons 2 to 11 (NM_000546.4)
Microsatellites: BAT25, BAT26, NR21, NR24, MONO27
